# Nanosecond-pulsed electroluminescence from high current–driven quantum-dot light-emitting diodes

**DOI:** 10.1126/sciadv.ads1388

**Published:** 2025-03-21

**Authors:** Tianhong Zhou, Fengshou Tian, Shuming Chen

**Affiliations:** Department of Electrical and Electronic Engineering, Southern University of Science and Technology, Shenzhen 518055, P. R. China.

## Abstract

Ultrashort optical emission, with pulse duration ranging from nanoseconds to femtoseconds, is usually obtained from lasers. In this work, we achieve nanosecond-pulsed electroluminescence (EL) from a solution-processed fast-response quantum dot light-emitting diode (QLED). By modeling the QLED with a resistor-capacitor equivalent circuit and analyzing the transient current of the circuit, the dynamic of the carrier injection and transport process that fundamentally affects the transient EL of QLED is revealed, which helps to guide the optimization of fast-response QLED. Driven by a high current source, the optimized QLED can output stable and repeatable ultrashort EL with a pulse duration of 20 nanoseconds, a repetition rate of 50 kilohertz, and a high radiant exitance of 5.4 watts per square centimeter. Enabled by the nanosecond-pulsed EL, the developed QLED can be directly used as an instantaneous excitation source for time-resolved fluorescence spectroscopy. Meanwhile, its use as an exposure flash for high-speed imaging is also demonstrated.

## Teaser

Fast-response quantum dot light-emitting diodes realize nanosecond-pulsed electroluminescence.

## INTRODUCTION

Ultrashort optical emission with high instantaneous power is of great interest due to its applications in ultrafast optoelectronics, ultrafast spectroscopy, high-resolution fast-imaging microscopy and materials processing (*1*–*6*). Usually, ultrashort emission with a pulse duration in the nanosecond to femtosecond range is obtained from lasers, which are quite expensive. To reduce the cost, there have been a few attempts to develop nanosecond-pulsed electroluminescence (EL) from inorganic III-nitride–based light-emitting diodes (LEDs) (*7*). By carefully designing the LED structures and driving the LED with a high current source, 10-ns-pulsed EL with a high repetition rate of 10 MHz has been realized (*8*). Such an ultrafast LED has been widely used as a low-cost excitation source for a wide range of spectroscopy applications (*4*, *9*). Recently, LEDs based on colloidal CdSe or InP quantum dots (QDs) have been hotly investigated as promising devices for display applications (*10*–*12*). Compared to LEDs, QD-LEDs or QLEDs are fabricated by solution processes, which offer unique advantages including lower cost, easier integration, higher flexibility, and larger area compatibility, making them highly attractive in some niche scenarios where LEDs cannot function properly (*13*–*15*). Typical QLEDs are based on an organic-inorganic hybrid structure, with inorganic ZnO nanoparticles commonly used as the electron transport layer (ETL) (*16*–*18*). The use of ZnO ETL offers a low electron injection barrier and high conductive electron transport, making the best-performing red QLED to exhibit a sub-bandgap turn-on voltage of 1.6 V (*19*), a maximum brightness of over 3,300,000 cd m^−2^ (*16*, *20*), and a theoretically high external quantum efficiency (EQE) of over 39% (*21*). Such excellent performance suggests that QLEDs are highly conductive, and thus they could respond to electrical stimulation as fast as LEDs. A few fast-response QLEDs have been developed and used for visible light communication, demonstrating a 3-dB bandwidth of 2.5 MHz (*22*). However, the best-performing fast-response QLEDs can only emit the microsecond-pulsed EL (*23*–*25*), and thus far, ultrafast QLEDs with nanosecond-pulsed emission have not yet been demonstrated.

To develop ultrafast QLEDs with nanosecond-pulsed emission, the transient EL (TREL) characteristics of QLEDs should be understood first (*26*). Upon the introduction of a voltage pulse, charge carriers rapidly inject into the devices and subsequently accumulate at the interfaces. For typical QLEDs, it is commonly believed that electrons are first injected into the QDs due to their relatively efficient injection and transport (*27*–*30*). The subsequent injection of the first hole into the QDs initiates the EL. The time it takes for the first hole to reach the QDs and recombine with an electron marks the EL delay time. As time passes, more holes reach the QDs, leading to an increase in EL. When most of the holes have been injected into the QDs and recombined with the electrons, the EL reaches its steady state (*31*). The time it takes for most holes to reach the QDs and recombine with the electrons determines the EL rise time. To realize ultrafast QLEDs, both the EL delay time and the rise time, which are determined by the hole transport paths and the hole velocity, should be as short as possible. However, the dynamics of the hole injection and transport processes, which fundamentally affect the TREL, are still unclear. Therefore, it is unclear how to optimize the devices to accelerate hole transport and recombination. Without understanding these fundamental processes, the realization of ultrafast QLEDs with nanosecond-pulsed emission is impossible.

In this contribution, we reveal the dynamics of the carrier injection, transport, and accumulation processes by studying the transient current (TRC) and the TREL of QLEDs. By modeling the QLED as a resistor-capacitor equivalent circuit and investigating the effect of functional layer thickness on the capacitance, the EL delay time, and the rise time, we disclose the charge accumulation interfaces and transport paths. With the understanding of these fundamental processes, we are able to specifically optimize the devices, and as a result, fast-response QLEDs with a short rise time of 10 ns are achieved. The optimized QLEDs can output stable and repeatable ultrashort EL with a pulse duration of 20 ns, a repetition rate of 50 kHz, and a high radiant exitance of 5.4 W cm^−2^ when driven by a high current source. We also demonstrate its use as an instantaneous excitation source for time-resolved fluorescence spectroscopy and as an exposure flash for high-speed imaging.

## RESULTS

### Charge carrier injection, accumulation, transport, and recombination dynamics

**Fig. 1.**
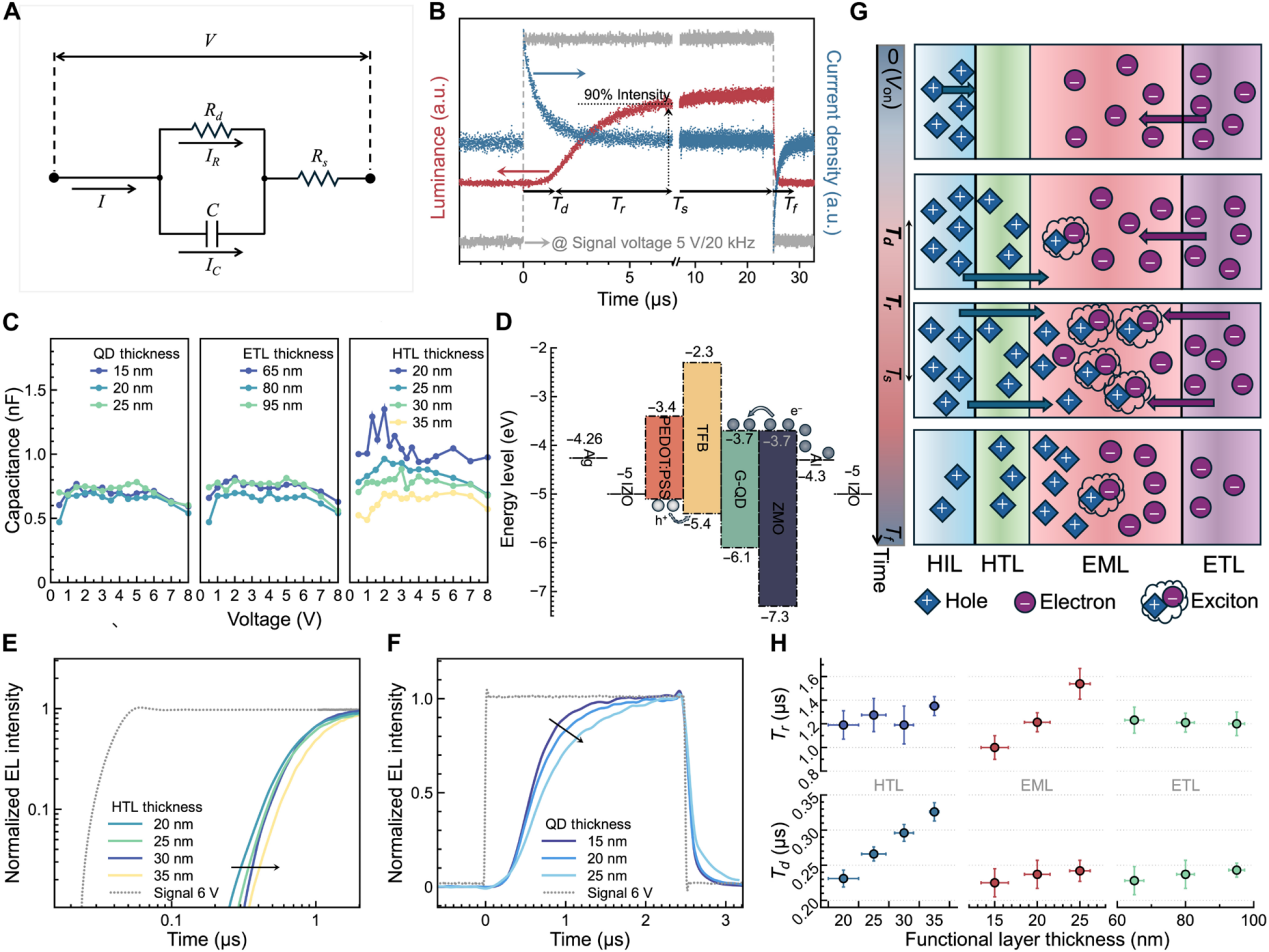
Charge carrier injection, accumulation, transport, and recombination dynamics. (**A**) Schematic diagram of the RC equivalent circuit of QLED. (**B**) Typical TRC and EL of a QLED biased by a 5-V pulse with a frequency of 20 kHz. The TREL is divided into four stages, characterized by the delay time (*T_d_*), the rise time (*T_r_*), the steady time (*T_s_*), and the fall time (*T_f_*). a.u., arbitrary units. (**C**) Calculated capacitance of QLEDs with different functional layer thicknesses. (**D**) Schematic diagram of the energy band levels of a top-emitting green QLED. The charges accumulated on both sides of TFB interfaces. (**E** and **F**) TREL of QLEDs with different HTL thickness (E) and QD thickness (F). (**G**) Schematic diagram of the charge injection, accumulation, transport, and recombination processes in QLEDs at different time intervals. (**H**) Statistical results of the rise and the delay time as a function of HTL, EML, and ETL thickness.

QLEDs are essentially p-n diodes and can thus be modeled as a resistor-capacitor equivalent circuit, as shown in Fig. 1A. The *R_s_* is the series resistor, which characterizes the resistance induced by the electrode leads and contacts. The *R_d_* represents the diode resistance, which is modulated by the drive current. The *C* is the junction capacitance caused by the space charge region. Figure 1B shows the TRC and EL of a QLED driven by a voltage pulse. The device initially functions as a capacitor, and thus on applying a voltage pulse, charges rapidly inject from the electrodes and charge the capacitor, resulting in a high charging current. Once the charging process is complete, the charge carriers transport and recombine in the QDs, leading to a steady recombination current and a stable EL. By fitting the TRC using (*26*)

$$I\left(t\right)=\frac{V}{R_{d}+R_{s}}\times \left(1+\frac{R_{d}}{R_{s}}e^{-\frac{t}{\tau }}\right)$$ (1)

$$\frac{R_{d}R_{s}}{R_{d}+R_{s}}C=\tau$$ (2)

the *C*, *R_s_*, and *R_d_* can all be extracted, as detailed in the Supplementary Materials (section S1). As shown in Fig. 1B, the TREL is closely related to the TRC and can be divided into four parts as characterized by time constants of *T_d_, T_r_*, *T_s_*, and *T_f_*, representing the EL delay time, rise time (defined as the time interval it takes the EL to go from 10 to 90% of its steady value), steady time, and fall time, respectively.

To investigate the charge accumulation process, the TRCs and ELs of the top-emitting QLEDs (glass/Ag/IZO/PEDOT:PSS/TFB/QD/ZnMgO/ultrathin Al/IZO) with different functional layer thicknesses were measured and fitted (figs. S1 to S3). As shown in Fig. 1C, the capacitance extracted from the fitting results remains almost the same as the thicknesses of QDs and ZnMgO ETL are varied, whereas it gradually decreases as the thickness of poly[(9,9-dioctylfluorenyl-2,7-diyl)-*alt*-(4,4′-(*N*-(4-butylphenyl)))] (TFB) hole transport layer (HTL) is increased. Such a result suggests that the TFB functions as the dielectric layer of the capacitor, and thus the charges are mainly accumulated on both sides of the TFB interfaces. This result can be understood by examining the energy level alignment of the QLEDs. As shown in Fig. 1D, the electrons can efficiently inject and transport to QDs due to the barrier-free interfaces. The injected electrons ultimately accumulate at the TFB/QD interface, whereas the holes mainly accumulate at the poly(3,4-ethylenedioxythiophene):poly(styrene sulfonate) (PEDOT:PSS)/TFB interface due to the presence of a 0.4-eV barrier. As a result, the capacitor is mainly affected by the thickness of TFB. To initiate the EL, the accumulated holes should transport through the TFB and recombine with the electrons in the QDs. The time it takes for the first hole to transit through TFB and recombine with an electron sets the *T_d_*. Therefore, the *T_d_* is influenced not only by the charging time but also by the thickness of the TFB as it determines the hole transit time. The *T_d_* is most sensitive to the thickness of the TFB (Fig. 1H, bottom), which gradually reduces as the thickness of the TFB decreases, as shown in Fig. 1E (fig. S4). After the EL is turned on, more holes reach the QDs, leading to the rise in EL. After most holes have injected into the QDs, they transport through the QDs to maximally overlap and recombine with the electrons in the QDs, resulting in a maximum and steady EL. Therefore, the *T_r_*, which characterizes the time it takes for most holes to recombine with the electrons, is mainly determined by the thickness of the QDs. As confirmed in Fig. 1 (F and H), the *T_r_* is mainly determined by the thickness of the QDs, which greatly decreases as the QD thickness is reduced.

The above findings enable us to draw a physical picture of the charge injection, accumulation, transport, and recombination processes in QLEDs. As schematically shown in Fig. 1G, upon the introduction of a voltage (*t* = 0), the charge carriers rapidly inject and charge the capacitor. At the end of charging, they accumulate on both sides of the TFB interfaces. At *t* = *T_d_*, the first hole has transited through the TFB and recombined with an electron in the QDs. As time passes by, more holes recombine with the electrons, resulting in an increasing EL. At *t* = *T_s_*, most holes have transited through the QDs and interpenetrated the electron-filling region, resulting in a maximum recombination rate and a maximum and steady EL.

The above model provides a theoretical basis for the optimization of fast-response QLEDs. It is obvious that the EL response is greatly affected by the *T_d_* and *T_r_*, which are mainly determined by the hole transit time through the TFB and QDs, respectively. Although the hole transit time can be decreased by reducing the thickness of the TFB and QDs, the device performance could be degraded, making this method impractical. By improving the hole mobility of both TFB and QDs, or by increasing the electric field dropped across both TFB and QDs, the hole velocity can be enhanced, and thus, the hole transit time can be shortened. In addition, the resistances of *R_s_* and ETL should be reduced so that the applied voltage is mainly dropped across the TFB and QDs to accelerate the drift of the holes. Both the enhanced hole velocity and reduced resistances of *R_s_* and ETL lead to an improved current. In other words, fast-response EL is usually achieved in high current–driven QLEDs. Therefore, to enhance the response of the QLED, we specifically focus on improving the current by reducing both the *R_d_* and *R_s_* of the QLEDs, as discussed below.

### The optimization of fast-response QLEDs

**Fig. 2.**
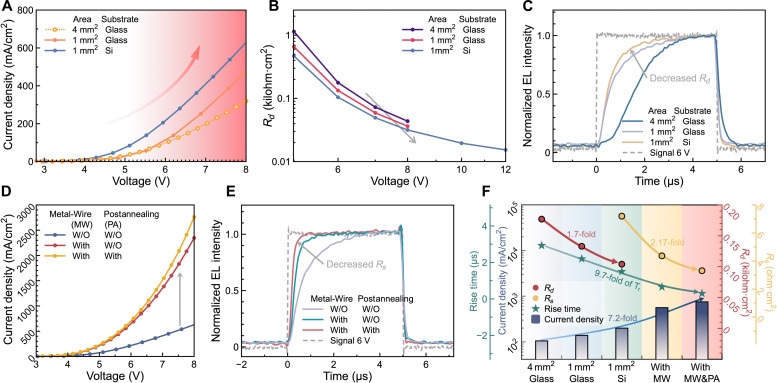
Optimization of fast-response QLEDs. (**A**) Current density (*J*)–*V* characteristics. (**B**) $R_{d}$-*V* characteristics ($R_{d}$ is normalized to emitting area). (**C**) TREL of devices with different areas and substrates. (**D**) *J*-*V* characteristics and (**E**) TREL of devices with/without metal wire auxiliary electrode or postannealing. (**F**) Effect of device structures on the *R_d_*, *R_s_* ($R_{s}$ is normalized to emitting area), current, and *T_r_*.

The area-normalized *R_d_* can be notably reduced by effective thermal management. As shown in Fig. 2A, a QLED with an emission area of 1 mm^2^ exhibits a notably higher current, especially at high voltage compared to that of the device with a 4-mm^2^ area. For example, at an applied voltage of 8 V, the 1-mm^2^ device shows a current of 461 mA cm^−2^, which is improved by 1.44-fold compared to 319 mA cm^−2^ of the 4-mm^2^ device, whereas at low voltage, both devices exhibit almost the same current. The voltage-dependent improvement suggests that Joule heat, which is largely generated at high voltage, should play an important role in affecting the current. This is reasonable because the Joule heat can promote the lattice vibration, and as a result, the charge carriers are scattered more strongly, thereby leading to the reduction in their mobility. By reducing the emission area, the Joule heat generated not only is reduced but also is effectively dissipated, thus resulting in an enhanced current. As shown in Fig. 2A, by further replacing the glass substrate with the Si, which has a higher thermal conductivity (*20*), the current is greatly improved, confirming that it is the Joule heat that affects the current. Figure 2B shows the *R_d_* that is extracted from the fitting results of the TRC (fig. S5). The QLED with a smaller area or a Si substrate shows a lower *R_d_*, which is in agreement with the *J*-*V* results. Because of the reduced *R_d_*, the devices can respond more quickly. For example, by reducing the area and using a Si substrate, the *T_r_* can be notably reduced from 2930 to 1500 ns at an applied voltage of 6 V, as shown in Fig. 2C.

The area-normalized *R_s_* can be effectively reduced by decreasing the resistances of both the electrodes and the contacts. Our top-emitting QLEDs use a 70-nm indium zinc oxide (IZO) as the transparent top electrodes, which exhibit relatively higher resistance and thus limit the improvement of current. By equipping the IZO top electrode with a peripherally auxiliary Al electrode (fig. S6), the resistance can be greatly reduced. As shown in Fig. 2D, with the Al peripherally auxiliary electrode, the QLED exhibits a current of 2347 mA cm^−2^ at 8 V, which is 3.7-fold higher than that of the control device. By further postannealing the device to promote the metallization between metal electrodes and semiconductors (*32*–*34*), the alloy contacts could be obtained, which not only reduce the contact resistance but also provide the barrier-free ohmic injection, both of which enable the current to be further improved to 2760 mA cm^−2^, as shown in Fig. 2D. As indicated by the fitting results of the TRC (fig. S7), such an improved current is ascribed to the reduced of *R_s_*. With a smaller *R_s_* and an enhanced current, the device responds faster. As shown in Fig. 2E, the *T_r_* is greatly reduced from 1500 to 300 ns by using a peripheral Al auxiliary electrode and postannealing the device.

Figure 2F summarizes the effect of device structures on the *R_d_*, *R_s_*, and current. By effective thermal management, the *R_d_* is greatly reduced by 1.7-fold, whereas by using the peripheral Al auxiliary electrode and postannealing, the *R_s_* is effectively reduced by 2.17-fold. With our dedicated device structures, both the *R_d_* and *R_s_* are gradually reduced, consequently enabling the current to be enhanced from 319 to 2760 mA cm^−2^ at 8 V, representing an enhancement of 8.7 times. Figure 2F also displays the relationship between the current and the *T_r_*. At an applied voltage of 6 V, when the current is enhanced from 105 to 753 mA cm^−2^, the *T_r_* is notably reduced from 2930 to 300 ns, showing an enhancement factor of 9.7-fold. It is obvious that fast-response EL is directly derived from high current–driven QLEDs. Therefore, to improve the response and realize nanosecond-pulsed EL further, the current should be enhanced.

### Nanosecond-pulsed EL from high current–driven QLED

**Fig. 3.**
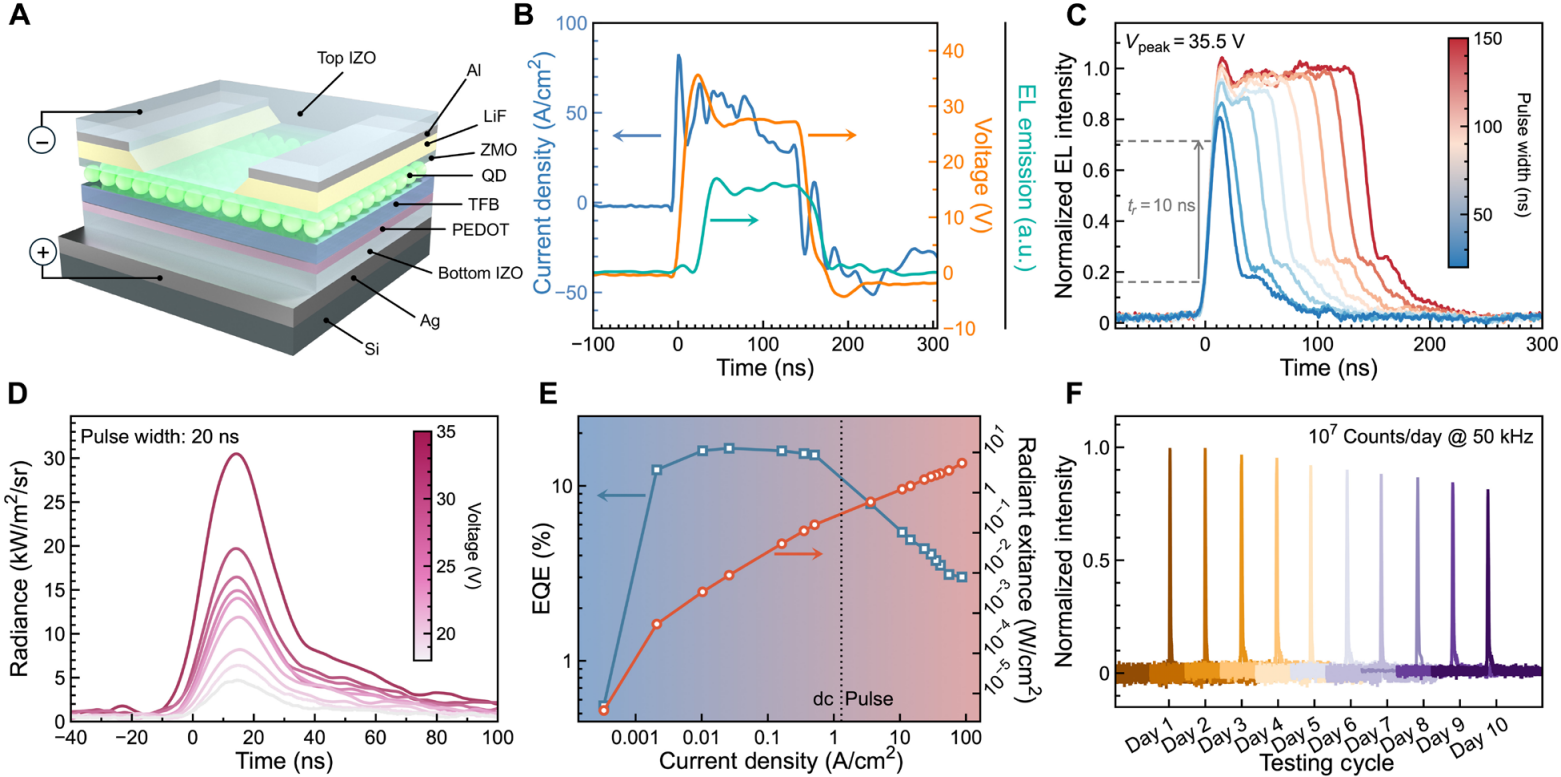
Nanosecond-pulsed EL from high current–driven QLED. (**A**) Schematic device structure of a fast-response QLED. (**B**) TRC and EL of QLED driven by a 35-V and a 150-ns voltage pulse. (**C**) TREL of the QLED driven by a 35-V (corresponding to a current density over 86 A cm^−2^) source with the pulse duration increasing from 20 to 150 ns. (**D**) TREL output of the QLED driven by a 20-ns voltage source with the voltage increasing from 18 to 35 V. (**E**) EQE and radiant exitance of the QLED as a function of current density. (**F**) Stability tests of nanosecond-pulsed EL from a QLED driven by a voltage source with a peak value of 35 V, a pulse duration of 20 ns, and a repetition rate of 50 kHz.

The schematic device structure of the final optimal fast-response QLED is shown in Fig. 3A. To further improve the current and realize ultrafast EL, the QLED was driven by a voltage pulse with a short pulse duration ranging from 20 to 150 ns (fig. S9A). At such a short pulse duration, the generated heat is limited and can be effectively dissipated, thereby enabling the QLED to operate normally, even at an extremely high current of up to 86 A cm^−2^. As shown in Fig. 3B, at a pulse duration of 150 ns and a voltage of 35 V, the QLED exhibits a high instantaneous current of 86 A cm^−2^ and rapidly responds to the applied voltage with a short *T_r_* of 10 ns. As shown in Fig. 3C, when the pulse duration is decreased from 150 to 20 ns, the device can still respond well to the applied voltage, as evidenced by the unchanged *T_r_*. Although the peak intensity of the 20-ns-pulsed EL is decreased to 82% of that of the 150-ns-pulsed EL, it is not due to the slow response of the QLED but rather to the lower voltage/current output of the source when the pulse width is reduced to 20 ns (fig. S9B). Actually, at a fixed pulse duration of 20 ns, the device can effectively respond to a wide range of voltages from 18 to 35 V (fig. S9C). With the increasing applied voltage, the output power of the 20-ns-pulsed EL is gradually increased, and a maximum output radiant exitance of 5.4 W cm^−2^ (corresponding to an instantaneous luminance of 2 × 10^7^ cd m^−2^) is obtained at an applied voltage of 35 V and a driven current of 86 A cm^−2^, as shown in Fig. 3 (D and E) and fig. S9D. The conversion between radiometry and photometry values of the green and blue QLED used in this study is shown in the Supplementary Materials (section S2 and table S1). Under such a high current driving, the EQE of the 20-ns-pulsed EL can still maintain a moderate value of 3%, which is lower than 15% of its continuous EL, as shown in Fig. 3E and fig. S10A. The low EQE of the nanosecond-pulsed EL is mainly ascribed to the increase in leakage current as well as the presence of Auger recombination and thermal-induced emission quenching that is triggered by the high current.

The fast-response QLED can output stable and repeatable 20-ns-pulsed EL with high power. As shown in Fig. 3F and fig. S10C, the device was continuously driven by a 35-V voltage pulse with a pulse duration of 20 ns and a repetition rate of 50 kHz. The device continuously outputs 10^7^ pulses per day with an initial fluence of 0.14 μJ cm^−2^ per pulse. After 10 days of operation, the EL intensity is only decreased to 80% of its initial value, indicating that the output nanosecond-pulsed EL is stable and repeatable. The developed fast-response QLED, with high instantaneous power and nanosecond-pulsed ultrashort EL, can be used as an instantaneous excitation source for laser pumping (*35*), photodynamic therapy (*36*–*38*), and ultrafast spectroscopy (*39*–*41*) or as a fast exposure flash for time-of-flight measurements (*42*–*44*) and high-speed imaging (*4*), as discussed below.

### Applications of nanosecond-pulsed QLEDs in time-resolved fluorescence spectroscopy and high-speed imaging

**Fig. 4.**
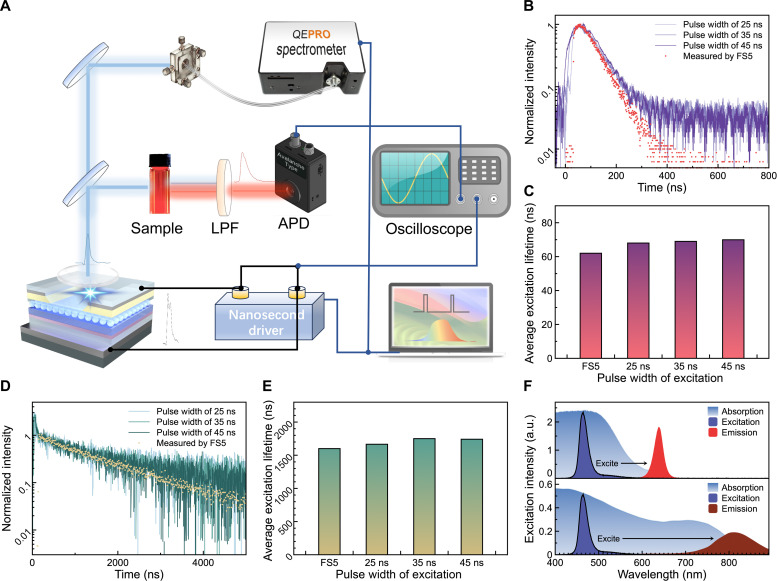
Applications of nanosecond-pulsed QLEDs in time-resolved fluorescence spectroscopy. (**A**) Schematic diagram of the homebuilt setup for TRPL spectroscopy. TRPL curves of (**B**) red QD and (**D**) infrared QD solution excited by a QLED with different pulse widths and commercial Edinburgh FS5 spectrofluorometer. Average exciton lifetime of (**C**) red QDs and (**E**) infrared QDs. (**F**) Absorption and PL spectra of the QD solutions and the EL spectra of a blue QLED excitation source.

As a proof-of-concept demonstration, the fast-response QLED with nanosecond-pulsed EL is used as an excitation source for time-resolved fluorescence spectroscopy, which is widely used to probe the excited state recombination dynamics (*45*). Figure 4A shows our homebuilt setup for the time-resolved photoluminescence (TRPL) spectroscopy. The sample (red or infrared QD solution) was excited by the nanosecond-pulsed blue QLED (fig. S11). Because of the excellent spectral overlap between the emission spectrum of the blue QLED and the absorption spectrum of the QDs (Fig. 4F), the QDs can be excited instantaneously and rapidly generate the PL. The generated time-dependent PL signal was detected by an avalanche photodiode (APD) and converted into a voltage signal, which was then recorded and displayed on an oscilloscope. A long-pass filter (LPF) was inserted between the APD and the QD sample so that only the PL of the QDs was detected. The excitation frequency was set at 2 kHz so that the period (500 μs) between two adjacent excitations is much longer than the lifetime scale of the QD sample. Figure 4 (B and D) shows the TRPL results of the QD samples excited by a QLED with different pulse widths. The results obtained from our homebuilt system agree well with those measured by a commercial Edinburgh FS5 spectrofluorometer (detailed setup shown in fig. S12), which uses a 65-ps-pulsed laser as an excitation source, indicating the reliability of our system. Because of the limited detection accuracy of the APD, there is a large noise when the PL intensity is decreased to 1/10th of its initial intensity. By replacing the APD with a single photon detector, as is used in FS5, the results obtained by our system should be highly similar to those obtained by FS5. To calculate the average exciton lifetime, the measured PL decay curves were fitted with an exponential function. As shown in Fig. 4 (C and E), the exciton lifetime obtained by nanosecond-pulsed QLED excitation is slightly longer than that obtained by FS5, and the difference decreases with decreasing excitation pulse width. Such a discrepancy is mainly caused by the different spectral ranges detected. Compared with FS5, which is equipped with a grating monochromator and can therefore only detect the PL signals at a specific wavelength, our system detects all the PL spectral signals, and thus the calculated lifetime represents the averaged lifetime of excitons at all wavelengths. The above results demonstrate that the low-cost QLED with nanosecond-pulsed EL can be a good substitute for expensive lasers for ultrafast spectroscopy applications.

**Fig. 5.**
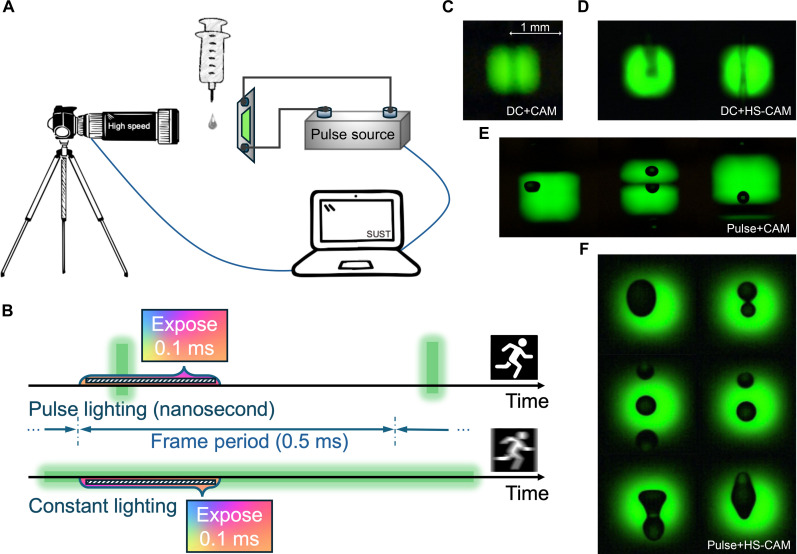
Applications of nanosecond-pulsed QLEDs in high-speed imaging. (**A**) Schematic setup of a homebuilt high-speed imaging system using a green QLED with a 20-ns-pulsed EL as the exposure flash. (**B**) Timing of the high-speed imaging with nanosecond-pulsed flash (top) and constant illumination (bottom). (**C** and **D**) With constant illumination, photographs of the fast-falling ink droplets captured by the conventional camera (C) and high-speed camera (D). (**E** and **F**) With 20-ns-pulsed flash, photographs of the fast-falling droplets captured by the conventional camera (E) and high-speed camera (F).

The ultrafast EL also enables the QLED to be used as a fast-exposure flash for high-speed imaging. High-speed photography is often used to capture images of fast-moving objects such as a falling water droplet, a speeding bullet, or a bursting balloon. Usually, high-speed photography is enabled by using a fast shutter, which can be faster than 1/1000th of a second, to freeze moments that are imperceptible to the naked eye. However, there is no such fast shutter that can capture the transient processes that occur in the nanosecond period. With the help of nanosecond-pulsed exposure flash, the objects are only exposed in the nanosecond period and therefore enable even a conventional camera to capture the nanosecond transient processes. Figure 5A shows our homebuilt system for capturing a fast-falling ink droplet. A green QLED with 20-ns-pulsed EL and a fluence of 0.14 μJ cm^−2^ is used as an exposure flash. A droplet ejector is fixed between the camera and the flash. To achieve a higher contrast, the ambient light is turned off during imaging. The repetition rate of the flash is set at 2 kHz to synchronize with the frame rate of the PHOTRON high-speed camera (1/2000 s) so that each frame only captures an exposure image. The camera’s shutter is set at 0.1 ms, which is much shorter than the frame time (0.5 ms). The detailed timing is shown in the top panel of Fig. 5B. With such a scheme, only the transient processes that occur in the exposure period (20 ns) can be recorded by the camera. Beyond the exposure period, even if the shutter remains open, the images cannot be recorded by the camera because there is no light to expose the objects. Compared to conventional photography with millisecond-pulsed flash or constant illumination (Fig. 5B, bottom), where the camera sensor is continuously exposed in the entire shutter period (0.1 ms or longer), thus blurring the ultrafast transient process, our method enables us to only freeze the moments occur in the nanosecond flash period, hence providing a clearer motion image.

As a demonstration, Fig. 5C shows the image of the falling ink droplets captured by a conventional camera or a high-speed camera (Fig. 5D) with constant illumination. The motion of a fast-falling droplet cannot be frozen by the sensor instantaneously (1/100 s for conventional camera and 1/10,000 s for high-speed camera), forming a blurring track. In this mode, the upper limit to record the fast movement of an object depends entirely on the shutter speed, as shown in Fig. 5B (bottom). By replacing the constant illumination with a 20-ns-pulsed flash, the motion of a fast-falling droplet can be rapidly frozen in the 20-ns period. A timescale of 20 ns is adequate to freeze almost arbitrary moving targets. With this method, even an ordinary digital camera can photograph what seems to be a continuous waterfall in an authentic and original moment of motion, as shown in Fig. 5E. By further using a high-speed camera, the instant of a falling droplet on a nanosecond timescale can be clearly captured, as demonstrated in Fig. 5F. The use of nanosecond-pulsed QLED flash contributes to unraveling the secrets of transient processes that occur in the nanosecond scale and extends the camera’s capability to ultrahigh-speed imaging.

## DISCUSSION

In summary, we reveal the dynamics of the charge injection and transport processes that fundamentally affect the response of QLEDs by analyzing the TRC of the resistor-capacitor equivalent circuit of QLEDs. The established physical model of the charge injection, accumulation, transport, and recombination processes provides a theoretical basis for the optimization of fast-response QLEDs. Guided by the model, the *R_d_* and the *R_s_* are specifically reduced, and as a result, the current is improved by 7.2-fold, which contributes to the realization of fast-response QLEDs with a short *T_r_* of only 300 ns at 6 V. By further driving the QLEDs using a high current source, the optimized QLEDs can output stable and repeatable ultrashort EL with a pulse duration of 20 ns, a repetition rate of 50 kHz, and a high instantaneous radiant exitance of 5.4 W cm^−2^. With our homebuilt systems, we showcase their practical applications as an instantaneous excitation source for time-resolved fluorescence spectroscopy and as an exposure flash for high-speed imaging. The developed solution-processed QLEDs, with ultrashort EL and high instantaneous power, could open up emerging applications for QLEDs in laser pumping, photodynamic therapy, ultrafast spectroscopy, time-of-flight measurement, and high-speed imaging.

## MATERIALS AND METHODS

### Materials

All materials were obtained from commercial companies. CdSe-based colloidal green/blue QDs (Cd*_x_*Zn_1−*x*_Se@ZnS, dissolved in octane solvent, 15 mg/ml) were purchased from Suzhou Mesolight Optoelectronics Co. Ltd. ZnMgO nanoparticles were obtained from Guangdong Poly Optoelectronics Co. Ltd. TFB was obtained from American Dye Source or VOLT-AMP Optoelectronic Technology Co. Ltd. PEDOT:PSS was obtained from Xi’an Polymer Light Technology Corp. Chlorobenzene, octane, and ethanol (99.99%) were purchased from Shanghai Aladdin Industrial Corp. IZO target (90 wt % In_2_O_3_ and 10 wt % ZnO) was provided by Hebei Gaocheng New Materials Technology Co. Ltd. Ultraviolet (UV) glue 6031 was obtained from NORLAND PRODUCTS Inc.

For time-resolved fluorescence spectroscopy: The red QDs and NIR-QDs bought from Suzhou Mesolight Optoelectronics Co. Ltd. used as a red and near-infrared fluorescence material were dissolved in octane solvent (15 and 0.5 mg/ml, respectively).

For high-speed imaging: The solution is black ink purchased from Deli Group Co. Ltd., and droplets were ejected from a homemade syringe with a 0.1-mm needle.

### Device structures

#### Top-emitting QLEDs

Glass/Ag (110 nm)/IZO-b (5 nm)/PEDOT:PSS (45 nm)/TFB (30 nm)/QDs (20 nm)/ZnMgO nanoparticles (80 nm)/Al (2 nm)/IZO-t (70 nm for green QLED and 50 nm for blue QELD).

#### Top-emitting QLEDs with auxiliary metal line electrode

Si (500 μm)/SiO_2_ (500 nm)/Ag (110 nm)/IZO-b (5 nm)/PEDOT:PSS (45 nm)/TFB (30 nm)/QDs (20 nm)/ZnMgO nanoparticles (80 nm)/Al (2 nm)/IZO-t (70 nm for green QLED and 50 nm for blue QELD). The insulating layer LiF (100 nm) and Al auxiliary metal electrode (80 nm) were evaporated on top of ZnMgO, with LiF defining the emitting area, as shown in Fig. 3A and fig. S6.

In the above structures, glass, Si/SiO_2_, Ag/IZO, PEDOT:PSS, TFB, QD, ZnMgO, Al (2 nm), and IZO-t function as a regular substrate, heat dissipation substrate, bottom electrodes, hole injection layers, HTLs, emission layers (EML), ETL, buffer layer, and top electrodes, respectively.

### Device fabrication

#### The substrate (glass or Si/SiO_2_) pretreatment

All substrates were soaked in an ultrasonic detergent for 30 min, followed by soaking in ultrasonic deionized water in an ultrasonic bath for 30 min and oven baking at 80°C for 60 min.

#### Bottom electrode

Stainless steel mask plates with patterns were used to define electrode patterns of 1 and 4 mm^2^. Ag [20–standard cubic centimeter per minute (SCCM) argon atmosphere, 50-W power] and bottom IZO (20-SCCM argon atmosphere, 50-W power) were deposited using magnetron sputtering with a processing pressure of 3 × 10^−1^ Pa and a power of 50 W. Before spin coating the functional layers, the Ag/IZO bottom electrodes were treated by an O_2_ plasma for 8 min. Then, the PEDOT:PSS (CLEVIOS P AI4083) was spun cast (3000 rpm, 45 s) and baked at 130°C for 30 min in the fume hood.

#### Hole transport layer/emission layer/electron transport layer

The substrates with Ag/IZO/PEDOT:PSS were then transferred into a nitrogen-filled glove box. Then, the TFB dissolved in chlorobenzene (at a concentration of 6, 8, 10, and 12 mg ml^−1^ for tuning the TFB thickness) was spun cast at 3000 rpm for 45 s, followed by baking at 120°C for 10 min. Green QD nanocrystals dissolved in octane (at a concentration of 6, 10, and 14 mg ml^−1^ for tuning the thickness of QD layers) or blue QD nanocrystals solution (10 mg ml^−1^ in octane solvent) was spun cast on the top of the TFB at 3000 rpm for 45 s and then baked at 100°C for 5 min. ZnMgO nanoparticles, dissolved in ethanol (at a concentration of 25, 30, 35, and 45 mg ml^−1^ for tuning the thickness) were spun cast onto the QD layer at 3000 rpm for 45 s and baked at 100°C for 15 min.

#### Top electrode

The samples were then transferred into a vacuum chamber (base pressure of 5 × 10^−4^ Pa) where ultrathin aluminum (2 nm) serving as a buffer layer was deposited at a rate of 0.8 Å s^−1^. Then, the samples were transferred into a magnetron sputtering system to deposit the IZO top electrode at an argon gas processing pressure of 0.3 Pa (20-SCCM argon atmosphere, 50-W power).

#### Metal auxiliary electrode

The auxiliary electrode with 100-nm LiF insulation layer and 80-nm Al layer was deposited by thermal evaporation with a deposition rate of 1.0 Å s^−1^ (base pressure of 5 × 10^−4^ Pa).

#### Encapsulation and postannealing

The QLEDs were encapsulated with UV resin and cover glass. Postannealing was performed at 90°C for 10 min using a hot plate.

### Device characterization

#### Thickness

The thicknesses of the Ag, IZO, PEDOT:PSS, TFB, ZnMgO, and QD films were measured by a Bruker DektakXT stylus profiler. The real-time evaporation rates and thicknesses of Al and LiF were in situ monitored by a quartz crystal microbalance.

#### Optical characteristic measurement

PL spectra, TRPL results, and excitation spectra were measured using the Edinburgh Instruments FS5 fluorescence spectrometer. The absorption of QD solutions was scanned by FS5, equipped with a cuvette. The central wavelength of PL is selected for measuring TRPL. The long-wave-pass filter (600-nm LP) used to measure the TRPL of the QD solution was a dielectric film filter mirror (Daheng Optoelectronics GCC-211104).

#### Steady photoelectric characterization

EL spectra of all QLEDs were collected by a fiber-optic spectrometer (USB-2000, Ocean Optics). *J*-*V*-*L*, EQE, and other optoelectronic properties were characterized by a dual-channel programmable source meter (Keithley 2614B) with a PIN-25D calibrated silicon photodiode. The emission solid angle of the QLED was measured by the Xi Pu Optoelectronics Angle Resolution Test System, which is subsequently used as an input parameter for the calculation of luminance.

#### Transient photoelectric measurement

For TREL and TRC testing, a dual-channel signal generator (Jun Ce Instruments, JDS6600) was used to output voltage signals (typically 6 V and 20 kHz) with one channel applied directly to the electrodes of the device and the other used as synchronization signal for the oscilloscope. The TREL signal was measured by an APD (Thorlabs APD110x). The QLED was series connected with a 50-ohm resistance for TRC testing. The voltage across the two ends of the resistor was converted to the TRC of the QLED device. Synchronization signals, as well as the TREL and TRC signals, are directly read by a four-channel high-precision digital fluorescence oscilloscope (TDS 3054C).

#### Nanosecond signal generator module

The Ultra-Narrow Pulse Module was custom developed, which can output adjustable nanosecond-pulsed electrical signal from 10 to 200 ns and 16 to 36 V. The output ports were soldered directly to the device electrodes to reduce transmission resistance, and the circuit board is equipped with a 50-milliohm resistor for TRC monitoring. The lifetime of optimized QLEDs under pulse mode was measured by a ZJZCL-2 tester, and the repetition frequency and pulse width were set at 50 kHz and 20 ns, respectively.

#### High-speed imaging

The Sony mirrorless camera NEX5N was used to photograph a droplet illuminated by a QLED flashlight operating in dc or pulse mode. The macro-optical lens (Venus Optics 65-mm f/2.8 E) was positioned at its minimum focusing distance to the droplets ensuring a maximum magnified image. The shutter speed in dc lighting mode was set to 1/50 s, ensuring that the CMOS (complementary metal-oxide semiconductor) was not overexposed, whereas the shutter speed in pulse mode was adjusted to 1/2000 s to avoid overexposure. The high-speed camera belongs to the Fastcam MINI AX series developed by Photron, and the macro-optical lens used was Nikon 90-mm f/2.8 Z. The frame speed was set at 1/2000 s, and the exposure time was set at exactly one frame time (1/2000 s).
