# Supplementary material for: Nanosecond-pulsed electroluminescence from high current–driven quantum-dot light-emitting diodes
Source: Sci Adv. 2025 Mar 21;11(12):eads1388. doi: 10.1126/sciadv.ads1388 (PMC11927609; doi:10.1126/sciadv.ads1388)
Supplement: Supplementary file 1 — Supplementary Text Figs. S1 to S12 Tables S1 and S2 [file sciadv.ads1388_sm.pdf]

Science Advances
AAAS

Supplementary Materials for

Nanosecond-pulsed electroluminescence from high current–driven quantum-dot light-emitting diodes

Tianhong Zhou *et al*.

Corresponding author: Shuming Chen, chen.sm@sustech.edu.cn

Sci. Adv. **11**, eads1388 (2025)
DOI: 10.1126/sciadv.ads1388

This PDF file includes:

Supplementary Text
Figs. S1 to S12
Tables S1 and S2

Supplementary Text

Section S1 The derivation process of R_d, R_s, and C in the equivalent circuit model

By fitting the transient current, the circuit parameters can be extracted. The following four equations can be inferred and expressed by the charge-defining equation, Kirchhoff's current law, the series voltage theorem, and the steady-state charge expression:

$$\int_0^t I_c(t)dt = Q_c \qquad eq.S(1)$$

$$I_R(t) + I_c(t) = I(t) \qquad eq.S(2)$$

$$R_d I_R(t) + R_s I(t) = V \qquad eq.S(3)$$

$$\big(R_d I_R(t)\big)C = Q_c(t) \qquad eq.S(4)$$

where I_R and I_C are the current flowing through R_d and C, respectively. $I(t)$ is the total current, R_d is parallel resistance and R_s is series resistance. We can use the first three expressions to obtain an expression for the total current, which is time-dependent:

$$I(t) = \frac{V}{R_d + R_s} \times \left(1 + \frac{R_d}{R_s} e^{-\frac{(R_d+R_s)}{R_d R_s C}t}\right) = \frac{V}{R_d+R_s} \times \left(1 + \frac{R_d}{R_s} e^{-\frac{t}{\tau}}\right) \qquad eq.S(5)$$

$$\frac{(R_d + R_s)}{R_d R_s C} = \frac{1}{\tau} \qquad eq.S(6)$$

Initially, the device functions as a capacitor, and thus, on applying a voltage pulse, charges rapidly inject from the electrodes and charge the capacitor, resulting in a high charging current of I_{max} that is defined by:

$$I_{max} = I(0) = \frac{V}{R_d + R_s} \times \left(1 + \frac{R_d}{R_s}\right) = \frac{V}{R_s} \qquad eq.S(7)$$

By substituting the I_{max} i.e., the maximum transient current, and the driving voltage V into *eq. (S7)*, the series resistor R_s can be obtained. Once the charging process is complete, the charge carriers transport and recombine in the QDs, leading to a steady recombination current of I_{stable} that is determined by:

$$I_{stable} = I(\infty) = \frac{V}{R_d + R_s} \qquad eq.S(8)$$

By substituting the steady current I_{stable}, the driving voltage V, and the series resistor $R_s$into *eq. (S8)*, the diode resistor R_d can be obtained. Finally, by inputting the R_s and R_d into *eq. (S5)* and fitting the transient current using *eq. (S5)*, the junction capacitance C can be obtained.

It should be noted that the C extracted from the transient current fitting results is somewhat different from the C conventionally measured by the semiconductor analyzer. Such a difference is caused by the difference in the driving signals (20 kHz square-wave signal vs DC voltage+1 kHz sinusoidal-wave signal). In conventional *C-V* testing, the device is pre-charged by a DC voltage, and the change of the accumulated charges is probed by an additional sinusoidal-wave signal; therefore, the obtained result reflects the steady state capacitance, allowing us to analyze the charge accumulation and recombination when the device is in steady state. In transient current testing, the device is directly driven by a high-frequency square-wave signal, and thus the fitting result reflects the transient state capacitance, which reflects the capacitance response to the moment of applied voltage. Therefore, the *C-V* results obtained from transient current are different from those obtained by conventional semiconductor analyzer.

Section S2 The conversion between radiometry and photometry values

Radiant flux (Φ_e, W) of the QLED can be directly measured by a calibrated photodiode (PD) combined with the emission spectrum of QLED. The radiant exitance (E_e, W/m^2) can be calculated by dividing the radiant flux by the emission area A. Radiance (L_e, W/m^2/sr) is determined by

$$L_e = \frac{E_e}{\Omega} = \frac{\Phi_e}{A \times \Omega} \qquad eq.(S9)$$

where Ω is the emission solid angle. The emission solid angle of a green QLED under ns-pulsed current excitation is determined to be 0.549π, as shown in fig. S8.

The luminance (L, cd/m^2) can be obtained by using the relationship between the radiometry and photometry:

$$L = K_m \int V(\lambda) L_e(\lambda) d\lambda \qquad eq.(S10)$$

where K_m is the maximum luminous efficacy, and $V(\lambda)$ is the luminous efficiency function. The calculated luminance for a green QLED and a blue QLED that are used in this study is shown in Table S1 and S2. At an ns-pulsed voltage of 34.5 V, the green QLED can output a maximum radiance of 5.4 W/cm^2, corresponding to a luminance of 2×10^7 cd/m^2.

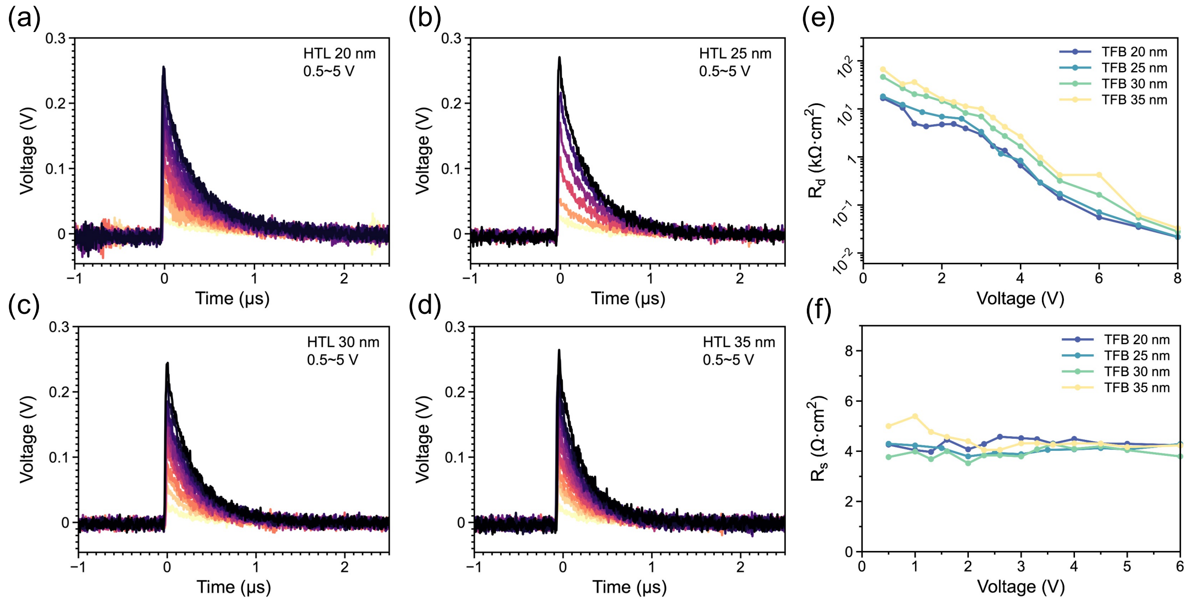

Fig. S1.

Transient currents and fitting results of QLED devices with different HTL thicknesses. HTL thickness is (**a**) 20 nm, (**b**) 25 nm, (**c**) 30 nm, (**d**) 35 nm. (**e**) Calculated R_d and (**f**) R_s with different HTL thicknesses as a function of voltage. All resistance is normalized to areas.

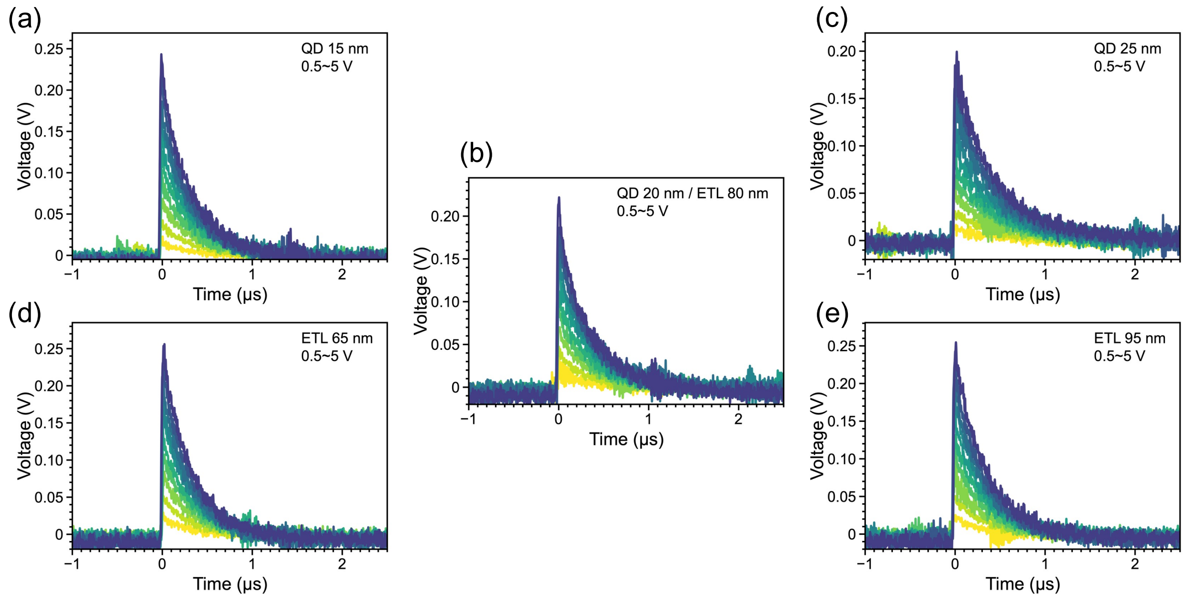

Fig. S2.

Transient currents of QLED devices with different EML/ETL thicknesses. EML thickness is (**a**) 15 nm, (**b**) 20 nm, and (**c**) 25 nm, respectively. ETL thickness is (**d**) 65 nm. (**b**) 80 nm and (**e**) 95 nm, respectively.

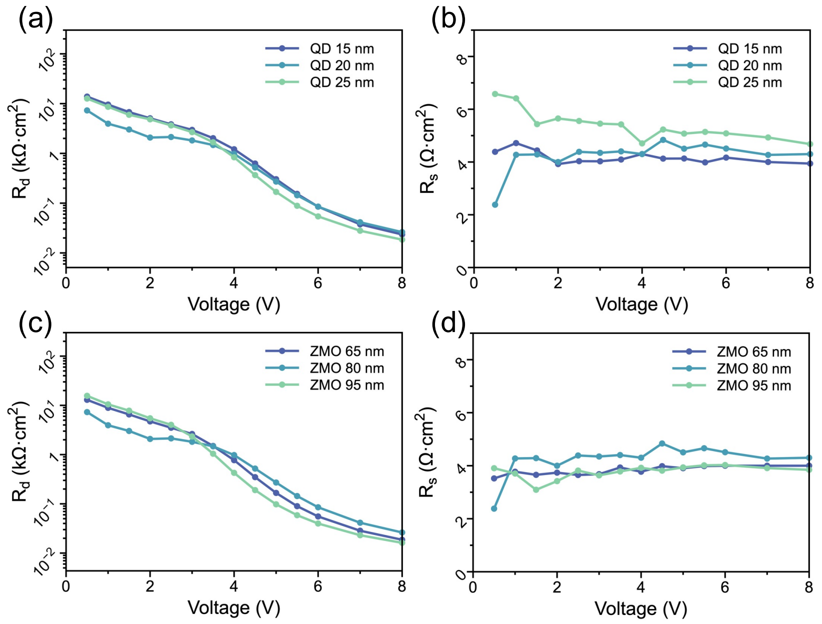

Fig. S3.

Fitting results of R_s and R_d of QLED devices with different EML and ETL thicknesses as a function of voltage. (**a**) Calculated R_d and (**b**) R_s with different QD thicknesses as a function of voltage. (**c**) Calculated R_d and (**d**) R_s with different ETL thicknesses as a function of voltage. All resistance is normalized to areas.

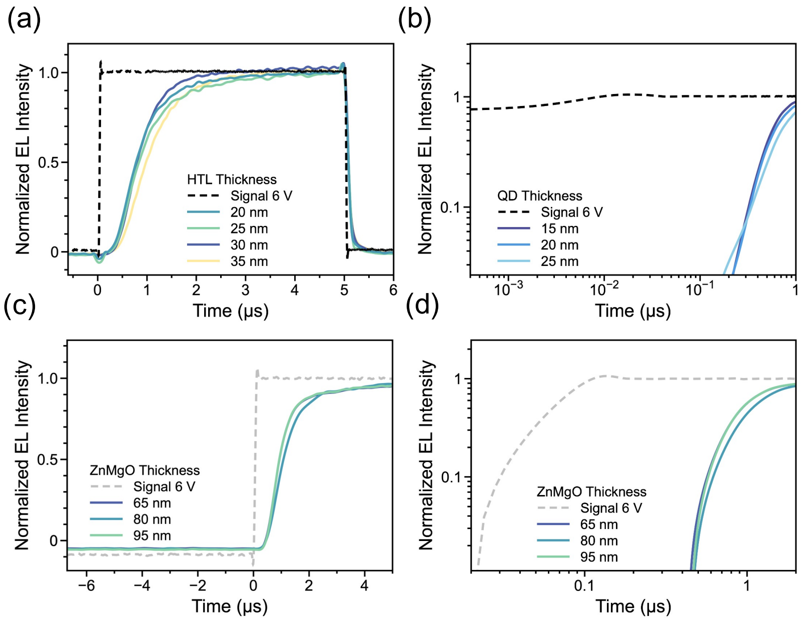

Fig. S4.

The transient EL of QLEDs with (**a**) different HTL thickness and (**b**) EML thickness. (**c**) Transient EL with varying ETL thickness with linear coordinates and **(d)** logarithmic coordinates.

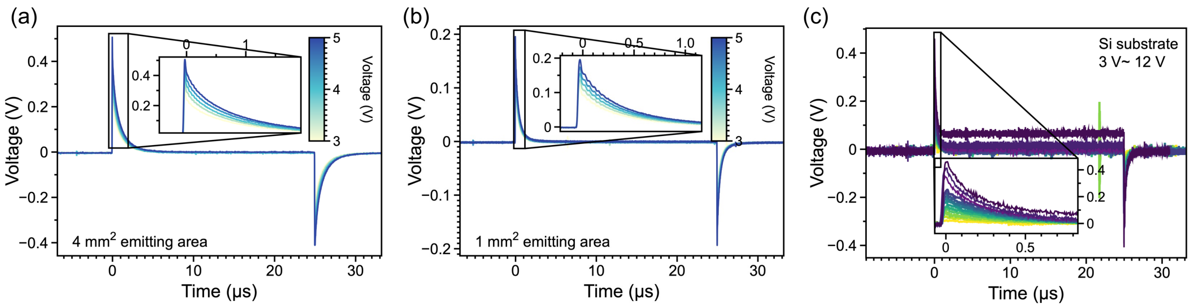

Fig. S5.

Transient currents of QLED devices with different emitting areas and substrates. (**a**) Transient currents of 4 mm^2 emitting area device and **(b)** 1 mm^2 emitting area device at the voltage range of 3-5 V. (**c**) Transient currents of 1 mm^2 emitting area device with Si substrate at the voltage range of 3-12 V.

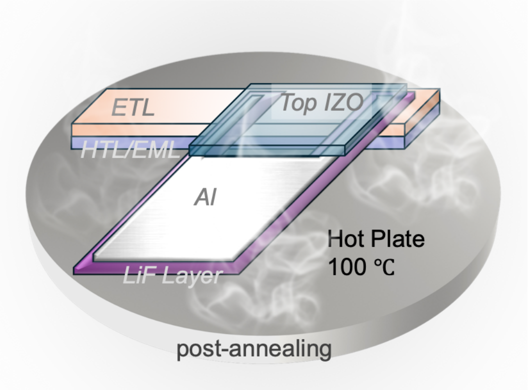

Fig. S6.

Schematic diagram of peripherally auxiliary Al electrode equipped with top IZO electrode. The aluminum electrode covers a slightly smaller area above the LiF insulating layer.

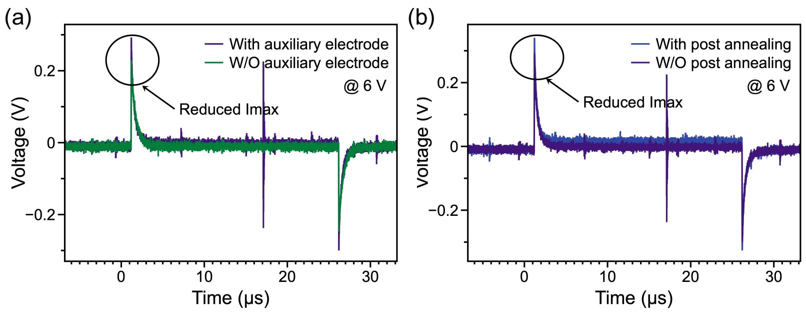

Fig. S7.

Transient currents of QLED devices with optimized *R_s*. (**a**) With/without metal wire auxiliary electrode. (**b**) With/without post-annealing at 6 V voltage bias.

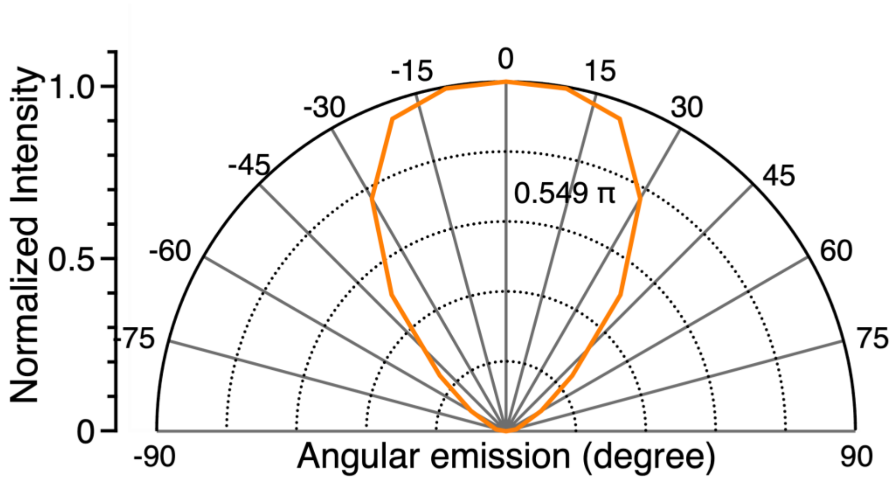

Fig. S8.

Angular emission profile of the green QLED driven by an ns-pulsed current source. The calculated emission solid angle is 0.549 π, which is used to calibrate the luminance value.

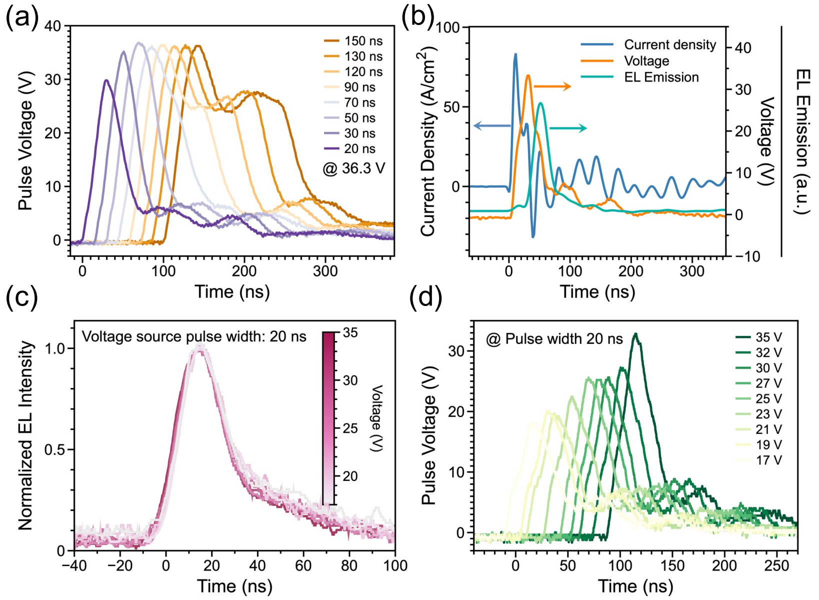

Fig. S9.

Transient current, voltage and EL of a green QLED driven by an ns-pulsed current source. (**a**) Transient output voltage pulse at different pulse widths. (**b**) Transient current, voltage and EL driven by a 35 V and 20 ns voltage pulse. (**c**) Normalized transient EL output of the QLED driven by a 20 ns voltage source with the voltage increasing from 17 to 35 V. (**d**) Transient output voltage pulse at different voltage amplitudes at the pulse width of 20 ns.

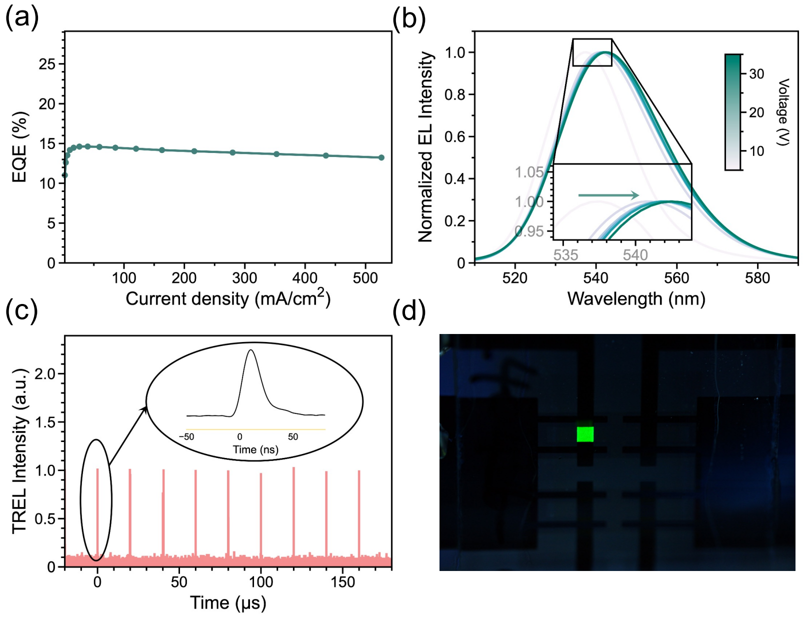

Fig. S10.

Green QLED device structure and performance (**a**) EQE of the green QLED. (**b**) Spectral redshift of the QLED from 5 V DC mode to 35 V pulse mode. (**c**) Global transient EL at 50 kHz repetition frequency; the inset is amplifying transient EL profile driven by a single voltage pulse. (**d**) Device photograph of transient EL in the stability test.

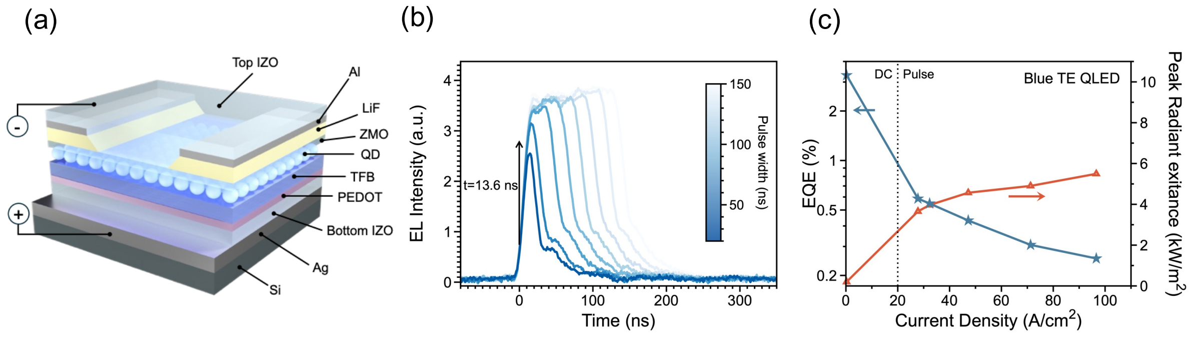

Fig. S11.

Blue QLED Schematic and Performance (**a**) Schematic diagram of device structure of a fast response blue QLED. (**b**) Transient EL of the blue QLED driven by a 35 V source with the pulse duration increasing from 20 to 150 ns. (**c**) EQE and radiant exitance of the QLED as a function of current density.

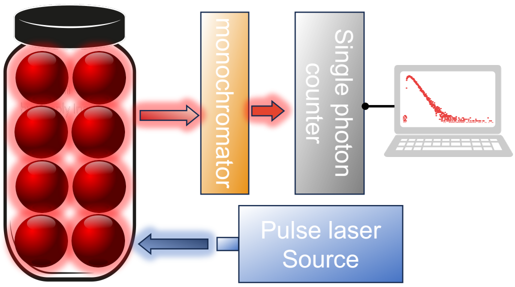

Fig. S12.

Time-resolved PL measurement setup based on commercial Edinburgh FS5 spectrofluorometer. The FS5 is equipped with a single photon counter for detecting weak signal intensity. The monochromator is used to filter emissions at specific wavelengths. The excitation source is a femtosecond pulsed laser with a central wavelength of 355 nm.

Table S1.

Radiance and luminance calculation of a green-QLED

	Voltage	Current	E_e	L_e	L	EQE
Unit	V	A/cm^2	W/cm^2	W/m^2/sr	cd/m^2	%
Pulse	34.5	8.6E+01	5.4E+00	3.1E+04	2.0E+07	3.0
	27.2	5.5E+01	3.6E+00	2.1E+04	1.3E+07	3.1
	25.6	4.1E+01	3.0E+00	1.7E+04	1.1E+07	3.5
	25.2	3.5E+01	2.7E+00	1.6E+04	9.8E+06	3.7
	24.8	3.0E+01	2.5E+00	1.4E+04	9.0E+06	4.1
	22.1	2.3E+01	2.1E+00	1.2E+04	7.6E+06	4.4
	20.0	1.4E+01	1.5E+00	8.5E+03	5.3E+06	4.9
	19.2	1.1E+01	1.2E+00	7.0E+03	4.4E+06	5.4
	17.2	3.6E+00	5.9E-01	3.4E+03	2.1E+06	7.9
DC	6	5.1E-01	1.6E-01	9.2E+02	5.7E+05	15.0
	5.5	3.5E-01	1.1E-01	6.5E+02	4.0E+05	15.3
	5	1.6E-01	5.3E-02	3.1E+02	1.9E+05	15.9
	4	2.6E-02	8.8E-03	5.1E+01	3.2E+04	16.4
	3.5	1.0E-02	3.4E-03	2.0E+01	1.2E+04	15.9
	3	2.1E-03	5.3E-04	3.1E+00	1.9E+03	12.3
	2.2	3.3E-04	3.8E-06	2.2E-02	1.4E+01	0.6

Table S2.
Radiance and luminance calculation of a blue-QLED

	Voltage	Current	E_e	L_e	L	EQE
Unit	V	A/cm^2	kW/m^2	W/m^2/sr	cd/m^2	%
Pulse	34.1	9.7E+01	5.5E+00	3.2E+03	4.5E+05	0.5
	25.4	7.1E+01	4.9E+00	2.8E+03	4.0E+05	0.6
	23.0	4.7E+01	4.6E+00	2.6E+03	3.7E+05	0.8
	19.1	3.2E+01	4.0E+00	2.3E+03	3.2E+05	1.0
	17.9	2.8E+01	3.7E+00	2.1E+03	3.0E+05	1.1
DC	6	2.7E-01	1.1E-01	6.4E+01	9.0E+03	3.3
